# Supporting Post-Stroke Language and Cognition with Pharmacotherapy: Tools for Each Phase of Care

**DOI:** 10.1007/s11910-023-01273-3

**Published:** 2023-06-05

**Authors:** Melissa D. Stockbridge, Zafer Keser

**Affiliations:** 1 Department of Neurology, Johns Hopkins University School of Medicine, 600 North Wolfe Street, Phipps 4, Suite 446, Baltimore, MD 21287 USA; 2 Department of Neurology, Mayo Clinic, Rochester, MN 55905 USA

**Keywords:** Pharmacotherapy, Adjunctive therapy, Neural plasticity, SSRI, Stroke, Aphasia

## Abstract

**Purpose of Review:**

There is enormous enthusiasm for the possibility of pharmacotherapies to treat language deficits that can arise after stroke. Speech language therapy remains the most frequently utilized and most strongly evidenced treatment, but the numerous barriers to patients receiving the therapy necessary to recover have motivated the creation of a relatively modest, yet highly cited, body of evidence to support the use of pharmacotherapy to treat post-stroke aphasia directly or to augment traditional post-stroke aphasia treatment. In this review, we survey the use of pharmacotherapy to preserve and support language and cognition in the context of stroke across phases of care, discuss key ongoing clinical trials, and identify targets that may become emerging interventions in the future.

**Recent Findings:**

Recent trials have shifted focus from short periods of drug therapy supporting therapy in the chronic phase to longer terms approaching pharmacological maintenance beginning more acutely. Recent innovations in hyperacute stroke care, such as tenecteplase, and acute initiation of neuroprotective agents and serotonin reuptake inhibitors are important areas of ongoing research that complement the ongoing search for effective adjuvants to later therapy.

**Summary:**

Currently there are no drugs approved in the United States for the treatment of aphasia. Nevertheless, pharmacological intervention may provide a benefit to all phases of stroke care.

## Introduction

Strokes impact millions of Americans and are projected to increase in prevalence by 20% over the next 10 years [1, 2]. However, a given individual’s needs after stroke vary widely as a function of the stroke extent and location. Post-stroke cognitive impairments have been estimated in as much as half of surviving adults [3, 4] and may include deficits in language, called aphasia. Treating aphasia is typically done with speech and language therapy (SLT) [5]. However, the process of rehabilitation therapy is long and arduous [6]. This has led to numerous lines of research into augmenting the effectiveness and efficiency of SLT hours, notably via neurostimulation [7–11] or pharmacological adjuncts [12, 13]. Enthusiasm among clinicians and patients alike for a “magic pill” to reverse ischemic brain injury’s effects has been reflected in reviews on the topic of pharmacology in aphasia [11–30], recently emerging at the rate of about one per year. Notably, the rate of reviews well exceeds that of clinical trials, positive or otherwise. Language capacities vary widely among diverse healthy adults and even more among adults in the post-stroke period, such that generating strong evidence of reliable benefits of pharmacological intervention when added to SLT is an enormous challenge, assuming those small effects truly exist at all. Null findings frequently are ascribed to ceiling effects associated with sufficiently intensive language therapy co-occurring with drug treatment, which suggests further large-scale clinical trials are needed to examine the interaction of pharmaceuticals and SLT dosage on language improvements [11]. To date, there remains no single pharmacological strategy that has generated sufficient evidence to justify mainstream therapeutic use. Even when investigations have been diverse and lengthy, as has been the case when considering levodopa [31–33] and bromocriptine [34–37], often when paired with therapy, there is no evidence that these result in better outcomes than placebo when used to support language after stroke at any phase of recovery. No medications are approved by the United States Food and Drug Administration (FDA) for the treatment of aphasia.

There is minimal evidence that pharmaceuticals are doing measurable harm to post-stroke aphasia severity or recovery either. In at least 80 years of inquiry, only a very limited list of pharmacological interventions has been identified as having potential deleterious effects on post-stroke recovery, namely dopamine antagonists [38–40], norepinephrine-dopamine reuptake inhibitors [41], benzodiazepines [41], and alpha blockers [15]. However, when underlying candidate mechanisms were examined in a longitudinal retrospective cohort of patients with left-hemisphere stroke, short term use of these drugs was not associated with poorer language outcomes [42], suggesting even these may be employed reasonably should the potential benefits outweigh the potential risks. These apparently conflicting findings highlight a changing aspect of pharmacotherapy in stroke rehabilitation. Early clinical trials primarily focused on brief, finite subscription windows [43, 44], but increasingly, studies have included longer windows of subscription [31, 45], approaching maintenance terms [46–50]. Many chronic conditions are treated with maintenance medications. Considering the long-term use of pharmacological candidates for maintenance of SLT-driven cognitive-linguistic gains allows previously dismissed therapies to gain a new potential for improving the lives of people with aphasia in ways that historically have been dismissed.

Rather than exclusively rehashing the relative paucity of evidence regarding SLT’s potential pharmacological adjuvants, this review reflects a conscious decision to take a step back and consider how pharmacotherapy may play a supportive role for language and cognition preservation and downstream rehabilitation from the moment of ischemia through chronic recovery. We hope this perspective will support a more immediately actionable, broader approach to considering the utility of these important tools for the benefit of our patients.

## Hyperacute

The first 24 h after symptom onset/last known well is considered the hyperacute period in stroke. The main purpose of interventions in this time window typically is to reverse the disease process and minimize the injury.

### Reperfusion Therapies in Ischemic Stroke

Intravenous thrombolytic agent, alteplase, has been the cornerstone of acute ischemic stroke therapy since 1995 [51]. However, a longer acting intravenous thrombolytic agent with potentially higher rates of revascularization, tenecteplase, has started to replace alteplase in acute stroke care [52]. In combination with mechanical thrombectomy, intravenous thrombolytics are powerful treatments that reduce hyperacute ischemic injury and improve outcomes. The most common outcome measure in hyperacute reperfusion therapy trials is the modified Rankin Scale (mRS), which is a functional outcome measure that robustly reflects ambulation. Language examination frequently is limited to the single broad qualitative assessment item on the National Institute of Health (NIHSS), upon which presence of aphasia is graded from absent to “mute” on a 0 to 3 scale. Thus, there is limited basis to examine the relationship between reperfusion therapy and aphasia directly. However, when the relationship has been evaluated [53–55], authors have found that these therapies contribute to aphasia recovery [54]. The indications of reperfusion are expanding. The main focus of innovation is to increase patient inclusion by extending the time window (NCT03785678) and loosening the stringency of the eligibility criteria [56]. Once the indications of reperfusion therapies are well established, in-depth and detailed evaluation of these therapies on aphasia can be evaluated more comprehensively.

### Prevention of Hematoma Expansion in Hemorrhagic Stroke

Hematoma expansion can aggravate acute hemorrhagic injury. Based on the preliminary positive evidence from smaller scale studies, recombinant factor VIIa (rVIIa) was tested in patients with intracerebral hemorrhage within the first 4 h in the phase III trial [57]. rVIIa significantly reduced hematoma growth but did not lead to an improvement in functional outcomes. Currently, the FASTEST trial is testing if rVIIa infusion would improve the outcome if infused within the first 2 h (NCT03496883).

## Acute

### Neuroprotective Agents

The main purpose of using neuroprotective agents is to mitigate secondary effects of the ischemia or hemorrhage such as excitotoxicity, neuroinflammation mediated through exposure to free radicals/reactive oxygen species (ROS), hemoglobin degradation products, or iron deposition to reduce the degree of vascular injury in the brain parenchyma. Although many agents were tested in pre-clinical stages, only key human studies will be highlighted here.

Uric acid is an antioxidant agent tested in acute ischemic stroke to reduce ROS-related injuries, which reduces infarct growth and improves mRS in patients with hyperglycemia or those receiving thrombolytic or mechanical thrombectomy [58–60]. Similarly, deferoxamine, an iron chelator and neuroprotective agent, ameliorates mRS trajectory in the long term [61]. It is unclear if beneficial effects would be seen specific to language. Replication in larger, multicenter trials is needed to ensure generalizability prior to introducing neuroprotection to the routine clinical practice in acute ischemic or hemorrhagic stroke. Meanwhile, riociguat, propylthiouracil, perphenazine (NCT05762146), hydrogen and minocycline (NCT03320018), verapamil (NCT03347786), and intra-arterial cold saline injection (NCT05032781) currently are under investigation.

### Induced Alteration of Blood Pressure

Systemic blood pressure is one of the main determinants of cerebral perfusion. In acute ischemic stroke, cerebral perfusion through collateral circulation becomes critical. Induced elevation of blood pressure theoretically may enhance circulation through collaterals [62]. Induced elevation of blood pressure was a popular intervention before the mechanical thrombectomy era, during which collateral circulation was enhanced through vasopressors, and this improved language functioning by reducing the amount of hypoperfused brain parenchyma [63]. Since mechanical thrombectomy became part of routine practice, induced elevation of the blood pressure fell out of favor. In patients with intracerebral hemorrhage, rapid normalization of high blood pressure with antihypertensives to prevent hematoma expansion is part of the routine clinical practice.

### Piracetam

Piracetam is a GABA derivative thought to modulate AMPA and NMDA receptors [64]. The largest randomized, double-blind study on piracetam in stroke to date was a negative trial based on results measured on the Orgogozo scale [65, 66]. However, patients who had aphasia (n = 373) were examined after the fact, and results were promising but not conclusive. Of those who received piracetam, 10% more had “recovered” from aphasia by 12 weeks versus placebo. Among those who received the dose within the first 7 h, there was an even larger difference between groups [44]. These findings support the notion that piracetam in the acute phase may benefit language recovery. Since then, one meta-analysis found no statistically significant effect of piracetam on aphasia severity [67], but did find evidence of improved written language specifically. It is possible that differences in outcome measures and duration of subscription could account for findings in favor of piracetam use in aphasia [68–70], particularly when combined with intensive SLT [71] to facilitate changes in perfusion [72].

### Selective Serotonin Reuptake Inhibitors

Selective Serotonin Reuptake Inhibitors (SSRIs) are a class of drugs that increase extracellular serotonin. In the United States, the class of drugs includes fluoxetine, sertraline, paroxetine, fluvoxamine, citalopram, escitalopram, and vilazodone. Approved indications include an array of mood and anxiety disorders. However, there has been considerable interest in the potential plasticity-supporting effects of increased serotonin on post-stroke recovery. Fluoxetine has been associated with positive effects on motor recovery in the acute phase (in the FLAME study, a large double-blind, placebo-controlled phase II trial) [73–76], but not when recovery was defined by courser measures (in the FOCUS study, a large double-blind, placebo-controlled trial in which full point improvement on the mRS was the outcome variable) [77]. Studies of SSRIs in acute stroke continue, with trials examining their utility in motor (NCT02767999) and language recovery ongoing [78].

## Subacute-Chronic

Pharmaceuticals in this phase of recovery often are considered with one of two rationales. First, there are those strategies employed to prolong or re-open periods of increased neural plasticity. These are thought to make the brain more susceptible to the hours of therapy at a cellular level. Second, there are those strategies that are thought to improve the likelihood that the patient is engaging with therapy to the best of their ability. These are thought to make the patient more receptive at a behavioral level. Ideally, one would imagine these two mechanisms occurring simultaneously. Patients would receive support that simultaneously increased spike timing dependent plasticity and made them optimally focused and engaged in therapy sessions.

Despite the limited evidence to date, we will highlight four candidates for adjunctive pharmacotherapy that have garnered considerable contemporary attention and appear to have the potential for further large-scale, double-blind trials. Based on a preponderance of the evidence, clinicians may find these to be appropriate to consider in the interim practice of medicine: dextroamphetamine, memantine, donepezil, and selective-serotonin reuptake inhibitors.

### Dextroamphetamine

Walker-Batson and colleagues investigated the effects of dextroamphetamine (D-AMP) on the recovery of aphasia in patients with subacute to early chronic ischemic stroke [79]. When combined with speech and language therapy, patients receiving D-AMP had greater recovery compared to placebo. In the subacute to the chronic stage, adequate blood pressure control is part of secondary stroke prevention care and D-AMP has not been associated with concerning elevation [29, 79, 80]. Small pilot studies that assessed safety and effectiveness of D-AMP combined with transcranial direct current stimulation [80] or donepezil, showed positive gains in language functions. However, larger randomized studies are needed to further elaborate on the efficacy of D-AMP alone or combined with other agents.

### Memantine

Memantine is a non-competitive NMDA receptor antagonist that increases the production of brain-derived neurotrophic factor (BDNF) [81], which in turn increases NMDA receptor activity, regulating synaptic plasticity in adults [82–86]. It is approved in the United States for the treatment of moderate to severe Alzheimer’s dementia [87]. However, there is interest in using it among adults with cognitive deficits of other etiologies, including chronic post-stroke aphasia. In a small randomized, double-blind, placebo-controlled, parallel-group trial (N = 28), 20 mg/day for 16 weeks resulted in a significant improvement in Western Aphasia Battery (WAB) performance (a measure of overall aphasia severity) over placebo [47].

Despite statistical significance, these differences were small (average of + 4.0 points, SE 0.7, versus + 0.8 points, SE 0.5 out of 100 possible points). To put this in context, WAB scores are interpreted in 25 point ranges, from the most severe (0–25) to the mildest (76–93.8) aphasia (performance above 93.8 is within functional limits for adults) [88]. Among those with chronic aphasia, the WAB has a high test–retest reliability, defined as 5.3 points/100 in mean absolute change [43]. Patients in both groups were moderately severe (51–75) at baseline (67.1/100 versus 65.8/100). Taken together, this suggests that while the improvement among those who received memantine was able to be measured, it may not be clinically meaningful for patients and could potentially have resulted from test–retest variation. Nonetheless, in a landscape where discernable improvements among those receiving candidate pharmacotherapies are rare, these findings are appreciated. Adding to the enthusiasm surrounding memantine is its profile of relative safety and tolerability, even when combined with other medications [89]. Larger trials of memantine are warranted to further determine the evidence supporting its use.

### Donepezil

The use of an acetylcholinesterase inhibitor, donepezil, for post-stroke aphasia therapy so far has been investigated in two randomized placebo-controlled trials. Berthier and colleagues [90] examined the effect of 12 weeks of 10 mg donepezil on overall aphasia severity and picture naming in 26 patients (13 per group) with chronic post-stroke aphasia. Immediately after washout, WAB scores were more improved among those who received donepezil. As in the memantine trial, though statistically significant, differences were negligible from the perspective of clinical interpretation and similar in scale to test–retest variability (average of + 6.4 points with a 95% confidence interval of 4.1 to 8.8 versus + 3.5 points with a 95% confidence interval of 1.9 to 5.2 out of 100 possible points). In the second randomized trial, Woodhead and colleagues [91] examined the effect of donepezil + SLT in 20 patients with chronic Wernicke’s post-stroke aphasia on speech comprehension. The authors found that when controlling for overall severity, patients performed more poorly when assessed after 5 weeks of 10 mg donepezil than placebo. Moreover, the interaction with severity and drug was significant—more severe patients experienced more negative change. The remaining studies regarding the use of donepezil for post-stroke aphasia is based on lower levels of evidence (grades 3a–4, including case–control studies, some of which are open label [92, 93] and case series/case studies [94, 95]), from which it is difficult to distill clinical recommendations. Although there is a clear justification for continuing to examine acetylcholine modulation in this population given the neurotransmitter’s purported role in language processing [for review: 96], what is not clear is whether donepezil is the appropriate agent of this change or whether modulation of acetylcholine will have a net positive effect on communication globally. There is a need for large-scale randomized controlled clinical studies, such as the upcoming investigation of donepezil combined with transcranial direct current stimulation (NCT04134416), meta-analyses, and comprehensive analyses of clinically meaningful differences.

### Selective Serotonin Reuptake Inhibitors

In addition to their use in the acute phase, there is further interest in the use of SSRIs to support chronic rehabilitation. Post-stroke depression is very common, impacting 1 in 3 stroke survivors, compared to 1 in 10–20 in the general population [97, 98]. It is possible that even this is an underestimate. Diagnosing depression in individuals with significant communication disabilities frequently is done based on vegetative signs, which may be misattributed or otherwise fail to be appropriately identified as symptoms of depression. Among those affected, the first episode typically takes place in the early chronic period, within the first year after stroke. Unfortunately, for most patients, this is also the period when rehabilitation services are most available and densely provided. Depression can directly impact cognitive–linguistic performance [99–103] and engagement with SLT [104].

While the possibility of depression is one rationale for their use, positive effects of SSRIs on post-stroke plasticity appear to be experience-dependent [105], augmenting the effects of SLT, and independent of SSRIs’ mitigation of post-stroke depression [106]. Although drugs that rely on other mechanisms of action to treat depression have been considered in stroke, positive findings appear unique to SSRIs. Six months of moclobemide, a monoamine oxidase inhibitor approved for the treatment of major depressive episodes, did not improve language performance in a randomized, double-blind, placebo-controlled trial, when initiated in the subacute period [107].

Among those *without* diagnosed mood disorders, fluoxetine appears to support motor recovery in the chronic phase [108]. There is mixed evidence of improvement in cognitive recovery [106], but changes are not captured in gross measures of disability [77, 109]. A recent Cochrane review found statistically significant benefits of SSRI in reducing dependency at the end of treatment, improving neurologic deficits and affect, but not overall measures of cognition [110]. An updated meta-analysis of 1549 patients found that SSRI use was associated with better overall recovery and functional independence [111].

Multiple studies have examined the use of SSRIs to support aphasia recovery specifically, with promising results. In a double-blind, randomized crossover trial, 10 patients with fluent post-stroke aphasia, receiving fluvoxamine for 4 weeks, improved in picture naming and had reduced perseverations [112]. Patients with left hemisphere ischemic stroke who received SSRIs from onset through recovery had significantly higher repetition scores than a sample matched in age, education, and time post-onset who did not receive SSRIs, despite the group who received the drugs having had larger infarcts on average [113]. Among those individuals, WAB Quartile in the chronic phase (interpretable for aphasia severity) was predicted education, volume of infarct, antidepressant use, and age. Continuous SSRI use for the first three months following stroke also has been associated with greater frequency of improved picture naming and inclusion of more elaborate content when describing a picture than those with similar initial aphasia severity, levels of depressive symptoms, lesion volume, and percentage of damage to important regions for language function [49].

## Concluding Remarks

We may never reach a time when prescribing drugs *for* the treatment of aphasia moves beyond the investigational and into the common practice. Reperfusion therapies in the hyperacute stage reverse the disease process and mitigate secondary injury that has the potential to cause aphasia. Although the beneficial effects of these therapies on aphasia rarely have been quantified, reducing global injury early on has a strong potential for improving cognition and language outcomes. Despite *prima facie* enthusiasm regarding the use of drugs to support recovery of language once aphasia occurs, there are numerous factors that must be considered when responsibly prescribing within this population. All pharmaceuticals come with risk–benefit considerations. Many of the substances discussed here are associated with significant dose-limiting adverse reactions [36, 114] that may impact comfort and quality of life. Limited means for communicating also means patients have limited capacity to reliably communicate discomfort. A further consideration is the prevalence of polypharmacy in the aging population. While recent work has considered the synergistic use of multiple drugs in post-stroke recovery [29, 115], polypharmacy contributes to an increased risk of medication error and poor compliance [116], and it should be avoided where unnecessary [15]. Finally, limited means for communicating also implies patients are not able to participate in nuanced discussions and evaluations of the options related to their care. When combined with potentially sparse vegetative signs of distress, there is a distinct ethical dimension to routinely treating or augmenting the established, effective treatment of aphasia—namely, SLT—with pharmacotherapy in the absence of overwhelming evidence of benefit or negligible appraised risk.

This is only the beginning. There is a broad array of twenty-first century therapies currently under investigation for their utility in support post-stroke rehabilitation, including growth factors [117, 118] and C–C chemokine receptor 5 inhibitors [119]. Combined with increased pursuit of precision medicine, there is an exciting future ahead for drug-supported stroke rehabilitation in the coming years [30], and addressing our patients’ communication needs will undoubtedly benefit from these ongoing innovations.
